# Draft genome of *Parageobacillus thermoglucosidasius,* a member of hydrogenogenic carbon monoxide utilizers, isolated from a freshwater lake sediment

**DOI:** 10.1128/mra.00795-23

**Published:** 2024-01-17

**Authors:** Shiho Nishida, Jota Suzuki, Masao Inoue, Ryoma Kamikawa, Takashi Yoshida

**Affiliations:** 1 Laboratory of Marine Microbiology, Graduate School of Agriculture, Kyoto University, Kyoto, Japan; 2 R-GIRO, Ritsumeikan University, Shiga, Japan; 3 College of Life Sciences, Ritsumeikan University, Shiga, Japan; Indiana University, Bloomington, Bloomington, USA

**Keywords:** CO metabolism, *Parageobacillus thermoglucosidasius*, carbon monoxide dehydrogenase, genome analysis, hydrogenogen, freshwater lake sediment

## Abstract

*Parageobacillus thermoglucosidasius* is a facultatively anaerobic thermophile and possesses carbon monoxide dehydrogenase and hydrogenase for carbon monoxide (CO) oxidation and hydrogen production, respectively. In this study, we report a draft genome of *P. thermoglucosidasius* isolated from a freshwater sediment, expanding our knowledge on the distribution of CO utilizers.

## ANNOUNCEMENT

*P. thermoglucosidasius* strains have been isolated from various environments, including a milk processing plant, a hot spring water sample, and a marine sediment (1–4).

The sediment at 72.6-m depth was collected from Lake Biwa in Japan (35° 13′ 13″ N, 135° 59′ 48″ E) on October 2021 using an HR-type core sampler (Rigo Co.). A 2.5 g sediment was incubated in 5 mL of the modified B medium for freshwater microorganisms (5) under CO:N_2_ (20:80) at 65°C for 1 day. In the modified B medium, MgSO_4_·7H_2_O, NaCl, and resazurin were depleted from the B medium (5). The 50 µL liquid phase was inoculated onto the NBRC802 agar medium and incubated at 65°C aerobically for 1 day. A single colony named strain B1-2 was picked up and resuspended in the modified B medium to investigate the CO oxidation activity of the strain. After 72 h of incubation under the aforementioned conditions, CO depletion (4.0 µmol/mL) and H_2_ and CO_2_ production (2.1 and 3.1 µmol/mL, respectively) were observed using gas chromatography (Shimadzu, Japan). The partial sequence of the 16S rRNA gene determined using a set of primers (B27f and U515r) (6, 7) exhibited 100% identity with that of *P. thermoglucosidasius* strain BGSC 95A1 (GenBank accession number: NR_043022.2), indicating that B1-2 belongs to *P. thermoglucosidasius*.

For genome sequencing, the isolate from frozen stock was grown on the NBRC802 medium. The DNA was extracted using a DNeasy blood and tissue kit (Qiagen, Valencia, CA, USA) following the manufacturer’s instructions. The genome library was prepared using MGIEasy FS DNA Library Prep Set (MGI Tech Co., Ltd.). Genome sequencing was performed by DNBSEQ-G400 2 × 200 bp (MGI Tech Co., Ltd. and Bioengineering Lab. CO., Ltd.), which generated 13,266,968 paired-end reads.

Default parameters were used for all software unless otherwise specified. Quality trimming and adapter removal of the generated reads were performed using Trimmomatic version 0.39 (8) with Phred score Q30, followed by assembling the filtered 7,876,982 paired-end reads with SPAdes version 3.15.4 (9). Annotation was performed with DFAST pipeline version 1.2.18 (10).

The draft genome of *P. thermoglucosidasius* B1-2 comprises 199 scaffolds, with a total length of 3,904,810 bp, an average genome coverage of 269×, *N*_50_ value of 199,687 bp, a GC content of 43.9%, and 3,795 open reading frames. The average nucleotide identity of B1-2 with *P. thermoglucosidasius* strains DSM2542^T^ and TG4, calculated using ANIb in pyani version 0.2.10 (11), was approximately 98.9% and 98.7%, respectively.

Consistent with the hydrogenogenic CO utilization, B1-2 possesses one copy of a gene cluster of carbon monoxide dehydrogenase/hydrogen-evolving hydrogenase complex, encoding 15 proteins as seen in strains DSM2542^T^ and TG4 (5, 12). Two circular plasmids were present in B1-2.

## Data Availability

The raw reads for paired-end sequencing of strain B1-2 were deposited in DRA under the accession number DRR495726. The draft genome sequence of *P. thermoglucosidasius* strain B1-2 was deposited in DDBJ under the accession number BTHJ01000000. Furthermore, the plasmid-derived sequences were deposited in DDBJ under the accession numbers LC777828 and LC777829.
